# ElliQ, an AI-Driven Social Robot to Alleviate Loneliness: Progress and Lessons Learned

**DOI:** 10.14283/jarlife.2024.2

**Published:** 2024-03-05

**Authors:** E. Broadbent, K. Loveys, G. Ilan, G. Chen, M.M. Chilukuri, S.G. Boardman, P.M. Doraiswamy, D. Skuler

**Affiliations:** 1 Department of Psychological Medicine, the University of Auckland, New Zealand; 2 Intuition Robotics, USA; 3 Durham Family Medicine, Duke University School of Medicine, USA; 4 Weill Cornell Medical College, USA; 5 Department of Psychiatry and the Center for the Study of Aging, Duke University, USA

**Keywords:** Loneliness, robots, older adults

## Abstract

**Background:**

Loneliness is a significant issue in older adults and can increase the risk of morbidity and mortality.

**Objective:**

To present the development of ElliQ, a proactive, AI-driven social robot with multiple social and health coaching functions specifically designed to address loneliness and support older people.

**Development/Implementation:**

ElliQ, a consumer robot with a friendly appearance, uses voice, sounds, light, and buttons through a touch screen to facilitate conversation, music, video calls, well-being assessments, stress reduction, cognitive games, and health reminders. The robot was deployed by 15 government agencies in the USA. Initial experience suggests it is not only highly engaging for older people but may be able to improve their quality of life and reduce loneliness. In addition, the development of a weekly report that patients can share with their clinicians to allow better integration into routine care is described.

**Conclusion:**

This paper describes the development and real-world implementation of this product innovation and discusses challenges encountered and future directions.

## Background

**T**he proportion of older people in the global population is steadily increasing (1). An estimated 1 in 5 people will be aged 60 years or older by 2050 globally, and by 2030 in the USA (2, 3, 4). However, the increases in lifespan are not necessarily accompanied by more years lived in health. Rather, the growing size of the older population places increasing strain on older care services, and innovations are urgently needed to augment capacity to deliver services.

Loneliness is a prominent health issue and describes a feeling of distress that occurs when the quality or quantity of one’s social relationships do not meet one’s perceived needs (5). It is a feeling of social isolation even when one is surrounded by other people (6). Older people are at high risk of loneliness due to changes in social structures and daily routines from adulthood, and declines in mobility and financial resources (7, 8). Loneliness significantly increases the risk of physical and mental morbidity (9) and has been associated with declines in cognitive capacity and increased frailty (9, 10) as well as mortality (11).

There are a variety of effective psychological treatments for loneliness, including Cognitive Behaviour Therapy, mindfulness, gratitude, social skills, reminiscence, social identity, and integrative approaches (12, 13). Therapies may be delivered in a one-on-one or group format, and may focus on reducing maladaptive social cognition, strengthening social support, increasing opportunities for social interaction, or teaching social skills (14). However, there may be issues with scalability and accessibility when they are delivered by a human therapist (15). Technological innovations can make loneliness interventions more accessible when designed appropriately. One promising technology is social robots.

Social robots are physically embodied artificial agents capable of interacting socially with users and are perceived as social entities (16). Social robots may reduce loneliness through direct interactions between the user and robot, through the robot serving as a social catalyst between people in an environment, or by the robot hosting video calls to loved ones (17). Social robots have been shown to improve loneliness and physical and mental well-being in older people in clinical studies (17, 18), including robots with capabilities driven by artificial intelligence techniques (19).

There are several advantages and disadvantages of the robots that have been tested for their effects on loneliness so far. Some of these robots are animal-like and function like pets (e.g. Paro (AIST, Japan), and Aibo (Sony, Japan)); they have interactive abilities, are commercially available, reliable, and have beneficial effects on loneliness. Although some have the advantage of being soft to touch and cuddle and provide comfort, they cannot converse with people, which limits their functionality. Other non-animal type robots were built for general purposes and been re-programmed for older adults (e.g. Nao (Aldebaran Robotics, France), iRobi (Yujin Robot Limited, Korea), Tiago (PAL robotics, Spain)). These robots are often good in small-scale research but are unsuitable for wide-scale deployment due to practical issues such as large size and cost, outdated hardware or software, or insufficient reliability for daily use outside of a research setting. Other robots are in their development and early testing phases, for example, Mario (MARIO Project Consortium, European Union). This research project is specifically aimed at designing robots for older adults, particularly for those with dementia; yet results are preliminary and the robot is not commercially available (20). A big issue is that even once robots have been developed and trialled, there is a big jump to successful commercialisation and an even bigger jump to adoption in clinical settings.

This paper describes a new robot called ElliQ (21), which was purpose-built to help combat loneliness and boost well-being in older adults. It is capable of conversation, available for rent and has technical support available. It has been deployed in real-world settings with promising results to date. This presents a different approach compared with most robots tested to date in that it has been implemented in large-scale community settings already, and the robot’s small size, low cost and the technical support provided may present a more viable solution to care providers looking for robotic companions at the current time. However, randomized controlled trials are needed to establish its efficacy compared to other forms of intervention, and more long-term data is needed. In the rest of this article, information about the development of ElliQ and real-world data is presented, along with challenges and future directions.

## The Robot

**Figure 1.**
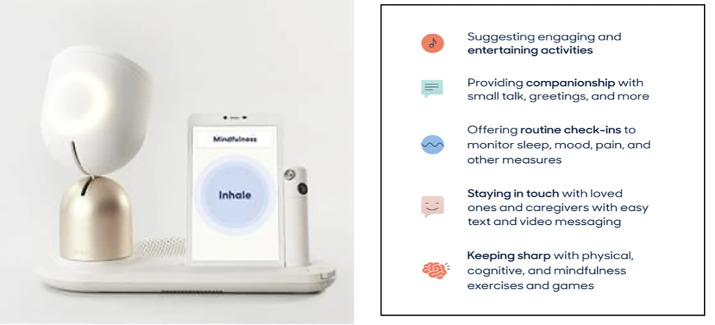
The ElliQ robot and the key activities it provides

Intuition Robotics was founded with the goal of developing an “active aging companion” technology to address social isolation and loneliness in the “silver” generation. ElliQ, a proactive, AI-driven companion robot, was launched by Intuition Robotics (Tel Aviv, Israel) in 2017 at the Design Museum in London. The company subsequently raised venture funding to support development, beta-testing, evidence generation, and commercial deployment. This social robot was specifically designed to address the unique challenges of the older population. Its character was designed to resonate culturally with US residents born 65 to 100 years ago. The content and activities were aimed at motivating and promoting greater independence as well as an active and socially connected life. ElliQ was designed to be proactive, empathetic, emotive, and embedded with multiple functions, including the ability to provide companionship and entertainment, health and wellness support, connection to loved ones, and assistance with daily activities (Figure 1).

(Intuition Robotics, Tel Aviv, Israel).

ElliQ is meant to be a consumer device and not a medical device. It is not intended to detect, diagnose, or treat medical conditions. However, the social relationship created between the user and the robot may help contribute to better health outcomes by providing stimulation, reminders for adherence, reducing loneliness and improving mood. The robot’s activities include conversation, music, video calls, well-being assessments, stress reduction, cognitive games, and reminders, among others (21). This tabletop, immobile robot uses voice, sounds, light, images, and buttons through a touch screen to interact with people. It was designed with a friendly, lamp-like appearance with an upper unit that swivels towards the user and lights up when conversing.

ElliQ initiates social interaction based on environmental sensing through a camera and microphone. It uses a proprietary AI algorithm to autonomously initiate and personalize suggestions and interactions based on the user’s learned personality, interests, and behavioral patterns. The algorithm takes into account factors including user behavior, sentiment analysis, preferences of previous interactions, and context in the day. ElliQ’s proactiveness and personalized suggestions for activities therefore dynamically change based on the user profile, current availability and conversational context, as well as identifying relevant interests and behavioral patterns of the user. With this in place, a relationship between ElliQ and the user evolves, and “trust“ is built. “Trust” within the relationship can be classified through frequency, variety, consistency and conversational depth of the user’s interaction with ElliQ. The software is updated every 3-4 weeks.

In 2018 and 2019, ElliQ underwent successful beta testing with about 200 users aged 62-97 years in California and Florida recruited through homecare agencies or directly, although these results were not published academically. The company collected data from usage patterns, user interviews, and in some cases, an external camera that recorded interactions (with user consent). Feedback from users who had it in their homes for at least six months revealed that many considered ElliQ a friend, rather than treating it as just another tech gadget. This finding is consistent with published studies of the relationships older adults form with other robots (17). Results of beta-testing allowed the company to “calibrate» the parameters of the algorithm, such as the frequency and nature of proactive behaviour, priority features and content offered, and the agent’s reactions to user interactions, and it was commercially launched in 2022.

## Implementation in Senior Care Settings

ElliQ was provided to older people across 15 programs from various healthcare organizations in the United States and Canada, including the New York Office for the Aging (NYSOFA), family medicine clinics, visiting nurse services, and Meals on Wheels. While some of the programs paid for the services, the older adults themselves did not pay nor receive payment for having an ElliQ at home. As part of the programs, users interacted with ElliQ from their homes to complete wellbeing activities and surveys. All usage and interaction data was collected for the purpose of research and product improvements. The ElliQ Privacy Policy and Terms of Service include consent for gathering and sharing the data with partners (in this case, the authors of this article).

Studies of real-world data and patient-rated outcomes enable an ecologically valid evaluation and may accelerate access to novel technologies for older patients who may benefit through the reduction of loneliness (22, 23). Initial metrics were collected from a subset of 173 users across these healthcare organizations, with at least 30 days of usage, who responded to a customer survey (24, 25). Survey responders had more active days using the robot compared to non-responders, although all users showed a relatively good degree of engagement. The response rate to the survey was 62% (173 of 278 users approached). Although this may be considered low by clinical trial standards, it is reasonable in this real-world context. On average, survey respondents had been registered with ElliQ for over nine months at the time of completing the survey. Respondents used the robot for an average of about seven months (use ranged from 17.9% to 100% of the days since registering).

**Figure 2.**
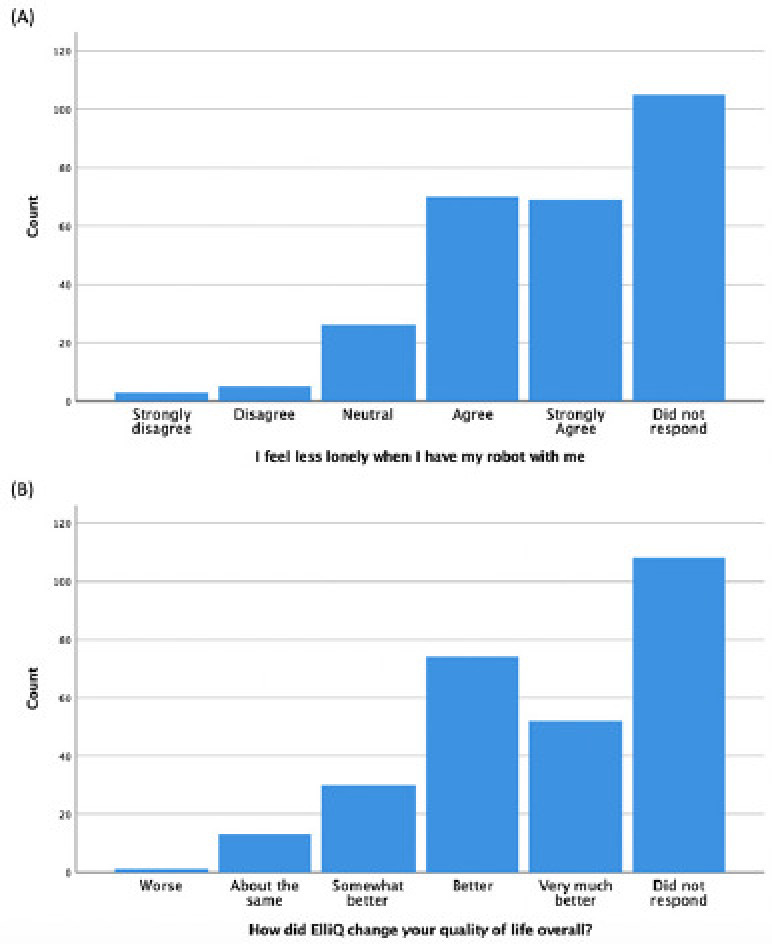
Survey results showing effects of ElliQ robot on users’ (A) loneliness and (B) quality of life in a pilot implementation

The ElliQ website reports real-world customer reports on how ElliQ changed their quality of life, loneliness, and well-being (25). 80% of respondents agreed or strongly agreed that they felt less lonely when the robot was with them, 74% said their quality of life was better or very much better since getting the robot (Figure 2), and this percentage increased to 90% when including those who said ‘somewhat better’. 56% agreed or strongly agreed that the robot helped them to connect to other people. While these numbers are promising, due to potential response bias, future research should follow up with the survey non-responders to understand whether their experience of using the robot differed.

Data is from Intuition Robotics company blog (21) – graphs are adapted with permission.

## Challenges

Social robots are a new category of products that are unfamiliar to seniors. One challenge was explaining and educating seniors about what ElliQ was and what it could do to help them in their daily lives. Some seniors were hesitant at first about welcoming a robot into their homes. In addition, some were surprised or annoyed by ElliQ’s proactive character, which prompts the users to engage in a conversation or activity several times a day. It was found that ElliQ is most suitable for older adults who live alone and don’t consider themselves socially active.

**Figure 3.**
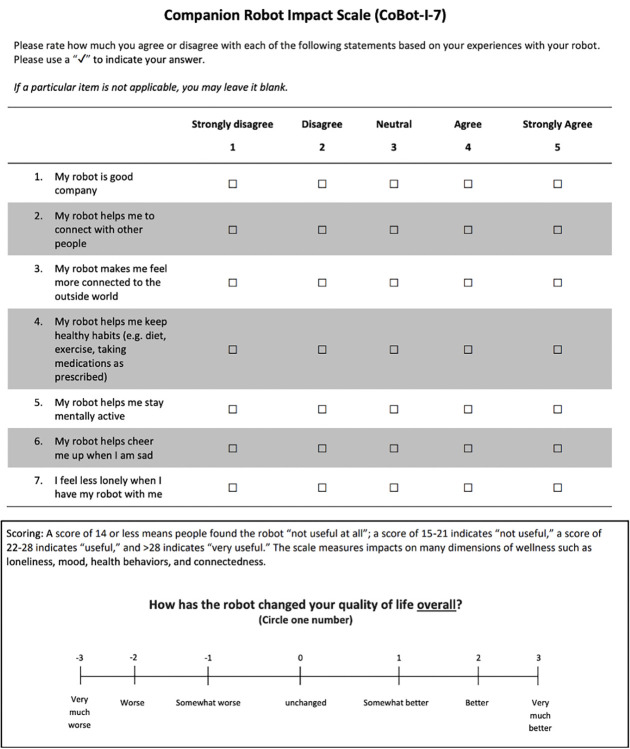
Companion Robot Impact Scale (CoBot-I-7)

Another challenge was how to assess the effects on loneliness and wellbeing. The Cobot-I-7 survey was selected as it has short, easy-to-understand questions and assesses loneliness, health, cognition, and mood. The last item assesses quality of life (see Figure 3). Alternative surveys were considered too complex and difficult to complete. The selected approach was for ElliQ to present the survey herself after at least 30 days of interaction with ElliQ at home to allow for possible companionship to evolve between the user and the robot.

Copyright 2022. It cannot be reproduced without permission from Dr Murali Doraiswamy.

**Figure 4.**
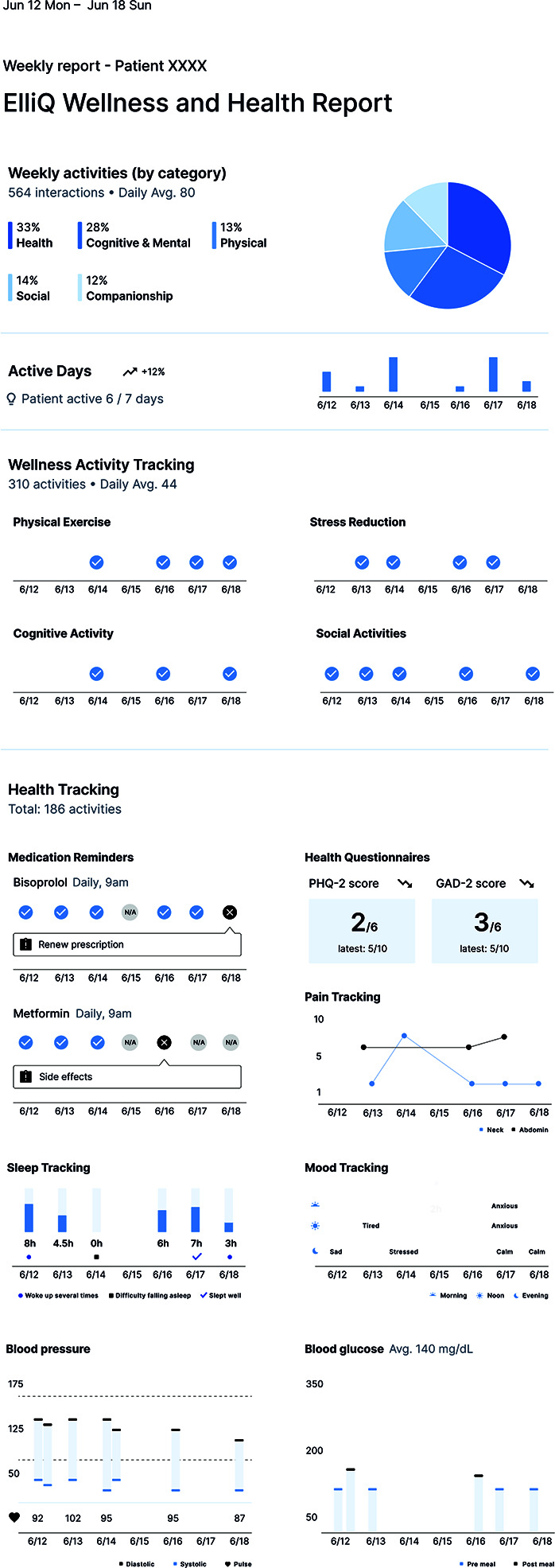
Example of an ElliQ patient usage report designed to be shared with a clinician

Users experienced some technological issues with the robot, requiring assistance. Therefore, real-world implementations must build in technical support phone lines and technicians who can make home visits. In addition, busy family physicians and geriatricians do not have time to integrate detailed daily dump reports from the robots for dozens of seniors into their daily schedules. A template for a summary weekly report that charts activity across several domains and highlights key areas for the physician to pay attention to was developed using feedback from a family practice (Figure 4). Physicians must also be reimbursed for the time they spend reviewing such information.

One challenge in the evaluation was that not all programs collected the same data (e.g., demographic information), limiting comparability and insights into the population. A data standardization exercise across the programs and standardized data on users’ physical, social, and mental activities would enhance future evaluations.

Data collection is ongoing, and the New York State Office for the Aging recently reported a 95% reduction in loneliness and meaningful improvement in well-being in their sample of 107 older adults (some of whom were a subsample of the 173 reported above and some were subsequent users) who had ElliQ for at least 30 days (26, 27). Engagement rates with the robot for users in the New York pilot were high (some 30 times per day on multiple days a week), with most of these interactions related to well-being. In the last year, several new features were added to ElliQ, such as guiding users with virtual museum tours or road trips, generative AI-based painting, mindfulness exercises, recording and sharing life memories, and the ability to send informational videos to users (27).

It is important to acknowledge some limitations of the robot. It is not mobile, so interactions are limited to where the robot is placed. It is not furry and huggable like a companion animal robot, and the robot’s conversations do not feel as natural as human-to-human conversations.

## Conclusion

The market for social robots is increasing, and the potential to reduce loneliness is becoming stronger as AI advances if designed appropriately (28, 29). Although social robots are consumer devices and are not intended to treat a specific medical condition, program evaluations should continue to collect real-world data on usage and effects in older people. In parallel, randomized controlled trials are needed to evaluate the efficacy and acceptability of social robots in this population compared to alternative loneliness interventions using validated assessments.

Adopting standardized ethical frameworks and outcome measures will significantly accelerate the field (29, 30). Potential unintended consequences of social robots and AI must be considered in the design and testing phases, and any negative outcomes must be monitored, so that risks can be mitigated. The novelty of this technology means that long-term outcome data is still emerging.

Target audiences for social robotic programs should include older adults living alone who are interested in inviting a social robot into their homes for 30 days or longer. A typical user may deal with one or more chronic illnesses and will require physical abilities, such as speech, hearing, and hand movement, to touch a screen. Intuition Robotics has set up a framework wherein healthcare organizations interested in initiating a program with ElliQ can contact them to mutually define its goals, boundaries, scale, and intended outcomes. Other robots such as Paro, iRobi, and AIBO have also been shown to benefit the loneliness and well-being of older people. They could be similarly used in programs with healthcare organizations and providers, although some of these robots are not currently commercially available. Virtual humans, especially those deploying large language models (generative AI), also have the potential to reduce loneliness, but more research and development on them is needed (17). Large-scale deployment of such agents in an ethical and evidence-based manner has the potential to help millions of older adults (29).

## References

[1] World Health Organization. Decade of healthy ageing: baseline report. Geneva, Switzerland: World Health Organization; January 14 2021. Accessed September 3 2023. https://www.who.int/publications/i/item/9789240017900

[2] Older people projected to outnumber children for first time in U.S. history. United States Census Bureau. March 13, 2018. Accessed July 19, 2023. https://www.census.gov/newsroom/press-releases/2018/cb18-41-population-projections.html

[3] World population ageing 2019: Highlights. New York, NY, United States: New York, NY: United Nations Department of Economic and Social Affairs; 2019. https://digitallibrary.un.org/record/3846855/files/WorldPopulationAgeing2019-Highlights.pdf

[4] World Health Organization. UN decade of healthy ageing: Plan of action 2021-2030. Geneva, Switzerland: World Health Organization; 2020. https://cdn.who.int/media/docs/default-source/decade-of-healthy-ageing/decade-proposal-final-apr2020-en.pdf

[5] Perlman D, Peplau L. Toward a social psychology of loneliness. In: Duck N & Gilmour R, eds. Personal Relationships in Disorder. London England: Academic Press, 1981:31-56.

[6] Cacioppo JT, Cacioppo S. The growing problem of loneliness. The Lancet. 2018;391(10119):426 10.1016/S0140-6736(18)30142-9 PMC6530780 29407030

[7] Chawla K, Kunonga TP, Stow D, Barker R, Craig D, Hanratty B. Prevalence of loneliness amongst older people in high-income countries: A systematic review and meta-analysis. PLoS One. 2021;16(7):e0255088. https://journals.plos.org/plosone/article?id=10.1371/journal.pone.0255088 34310643 10.1371/journal.pone.0255088 PMC8312979

[8] Davies K, Maharani A, Chandola T, Todd C, Pendleton N. The longitudinal relationship between loneliness, social isolation, and frailty in older adults in England: a prospective analysis. The Lancet Healthy Longevity. 2021;2(2):e70-e7. 36098160 10.1016/S2666-7568(20)30038-6

[9] Ong AD, Uchino BN, Wethington E. Loneliness and health in older adults: a mini-review and synthesis. Gerontology. 2016;62(4):443-9. https://karger.com/ger/article/62/4/443/147575/Loneliness-and-Health-in-Older-Adults-A-Mini 26539997 10.1159/000441651 PMC6162046

[10] Gale CR WL, Cooper C. Social isolation and loneliness as risk factors for the progression of frailty: the English Longitudinal Study of Ageing. Age Ageing. 2018;47(3):392-7. 10.1093/ageing/afx188 29309502 PMC5920346

[11] Holt-Lunstad J, Smith TB, Layton JB. Social relationships and mortality risk: a meta-analytic review. PLoS Med. 2010;7(7):e1000316. 10.1371/journal.pmed.1000316 20668659 PMC2910600

[12] Masi CM, Chen HY, Hawkley LC, Cacioppo JT. A meta-analysis of interventions to reduce loneliness. Pers Soc Psychol Rev. 2011;15(3):219-66. 10.1177/1088868310377394 20716644 PMC3865701

[13] Hickin N, Käll A, Shafran R, Sutcliffe S, Manzotti G, Langan D. The effectiveness of psychological interventions for loneliness: A systematic review and meta-analysis. Clin Psychol Rev. 2021;88:102066. 34339939 10.1016/j.cpr.2021.102066

[14] Cacioppo S, Grippo AJ, London S, Goossens L, Cacioppo JT. Loneliness: Clinical import and interventions. Perspect Psychol Sci. 2015 Mar;10(2):238-49. 10.1177/1745691615570616 25866548 PMC4391342

[15] Lavingia R, Jones K, Asghar-Ali AA. A systematic review of barriers faced by older adults in seeking and accessing mental health care. J Psychiatr Pract. 2020;26(5):367-82. 10.1097/PRA.0000000000000491 32936584

[16] Naneva S SGM, Webb TL, Prescott TJ. A systematic review of attitudes, anxiety, acceptance, and trust towards social robots. Int J Soc Robot. 2020;12(6):1179-201. 10.1007/s12369-020-00659-4

[17] Gasteiger N, Loveys K, Law M, Broadbent E. Friends from the future: A scoping review of research into robots and computer agents to combat loneliness in older people. Clin Interv Aging. 2021:941-71. 10.2147/CIA.S282709 PMC8163580 34079242

[18] Pu L, Moyle W, Jones C, Todorovic M. The effectiveness of social robots for older adults: A systematic review and meta-analysis of randomized controlled studies. Gerontologist. 2019;59(1):e37-e51. 10.1093/geront/gny046 29897445

[19] Loveys K, Prina M, Axford C, et al. Artificial intelligence for older people receiving long-term care: a systematic review of acceptability and effectiveness studies. Lancet Healthy Longev. 2022;3(4):e286-e97. 10.1016/S2666-7568(22)00034-4 35515814 PMC8979827

[20] Mario project. 2015. Accessed February 8 2024. http://www.mario-project.eu/portal/

[21] Intuition Robotics. How is ElliQ different from other devices on the market? Tel Aviv, Israel: ElliQ; 2023. Accessed September 3 2023. https://elliq.com/pages/features#.

[22] Framework for FDA’s real-world evidence program. US Drug and Food Administration. December 2018. Accessed 19 July 2023. https://www.fda.gov/media/120060/download

[23] Focus area: Patient-reported outcomes and other clinical outcome assessments. US Drug and Food Administration. 9 June 2022. Accessed July 19 2023. https://www.fda.gov/science-research/focus-areas-regulatory-science-report/focus-area-patient-reported-outcomes-and-other-clinical-outcome-assessments

[24] NYSOFA Deploys ElliQ Care Companion to Reduce Battle Social Isolation in Older Adults The Journal of mHealth 2022. June 1 2022. Accessed September 3 2023 https://thejournalofmhealth.com/nysofa-deploys-elliq-care-companion-to-reduce-battle-social-isolation-in-older-adults/.

[25] Intuition Robotics. The Results Are In - Measuring the Efficacy of ElliQ. August 16 2023. Accessed 3 September 2023. https://blog.elliq.com/measuring-the-efficacy-of-elliq

[26] New York State Office for the Aging. NYSOFA’s Rollout of AI Companion Robot ElliQ Shows 95% Reduction in Loneliness. August 1 2023. Accessed September 4 2023. https://aging.ny.gov/news/nysofas-rollout-ai-companion-robot-elliq-shows-95-reduction-loneliness .

[27] ElliQ. Engagement data from ElliQ users who received their device through the New York State Office for the Aging. August 1 2023. Accessed September 4 2023. https://aging.ny.gov/system/files/documents/2023/08/nysofa-and-elliq-engagement-report-july-2023.pdf

[28] Grand View Research. Healthcare Companion Robots Market Size, Share & Trends Analysis Report By Type (Animal-like, Humanoid), By Age-Group (Children, Adult, Geriatric), By Region, And Segment Forecasts, 2023 – 2030. Accessed September 3 2023. https://www.grandviewresearch.com/industry-analysis/healthcare-companion-robots-market-report

[29] Broadbent E, Billinghurst M, Boardman SG, Doraiswamy PM. Enhancing social connectedness with companion robots using AI. Sci Robot. 2023;8(80):eadi6347. https://www.science.org/doi/abs/10.1126/scirobotics.adi6347 37436971 10.1126/scirobotics.adi6347

[30] Ashok M, Madan R, Joha A, Sivarajah U. Ethical framework for Artificial Intelligence and Digital technologies. International Journal of Information Management. 2022;62:102433.

